# Supplementary material for: Secreted Glycosyltransferase RsIA_GT of Rhizoctonia solani AG-1 IA Inhibits Defense Responses in Nicotiana benthamiana
Source: Pathogens. 2022 Sep 9;11(9):1026. doi: 10.3390/pathogens11091026 (PMC9501517; doi:10.3390/pathogens11091026)
Supplement: Supplementary file 1 [file pathogens-11-01026-s001.zip › pathogens-1880179-supplementary-Table S1.pdf]

Table S1. Primer sequences used in this study.

No.	Primer name	Sequence: 5′- 3′	Remarks
1	RsIA_GT-F	ATGCCAACCCCGGGCCCA	Forward primer to amplify *RsIA_GT*
2	RsIA_GT-R	CTTTTGTGTAGCGTCCACAACTCG	Reverse primer to amplify *RsIA_GT*
3	Nb-RbohB-F	TCACAAGAGCTCAGGCGTTT	Forward primer for RT-PCR of *N. benthamiana RbohB* gene
4	Nb-RbohB-R	TCATCGAACCGCTTCTCGAC	Reverse primer for RT-PCR of *N. benthamiana RbohB* gene
5	Nb-WRKY12-F	CTCATCAGCTAGTTCATTTGATGC	Forward primer for RT-PCR of *N. benthamiana WRKY12* gene
6	Nb-WRKY12-R	AGCTCGGTCTTTGTTCTAAAAGC	Reverse primer for RT-PCR of *N. benthamiana WRKY12* gene
7	Nb-PR4a-F	CAACCCACAGAACATTAACTGG	Forward primer for RT-PCR of *N. benthamiana PR4a* gene
8	Nb-PR4a-R	TTGTCGGCATCCCAAGTAGT	Reverse primer for RT-PCR of *N. benthamiana PR4a* gene
9	Nb-EF1α-F	AGAGGCCCTCAGACAAAC	Forward primer for RT-PCR of *N. benthamiana EF1α* gene
10	Nb-EF1α-R	TAGGTCCAAAGGTCACAA	Reverse primer for RT-PCR of *N. benthamiana EF1α* gene
11	RsIA_GT-F1	TGGTACTTTCACCCTCCATACCAAC	Forward primer for signal peptide validation of RsIA_GT in *N. benthamiana*
12	RsIA_GT-R1	GTTGGTATGGAGGGTGAAAGTACCA	Reverse primer for signal peptide validation of RsIA_GT in *N. benthamiana*
13	RsIA_GT-F2	ATGCCAACCCCGGGCCCA	Forward primer for signal peptide validation of RsIA_GT in *N. benthamiana*
14	RsIA_GT-R2	TAGCGACGCACACGTAGACG	Reverse primer for signal peptide validation of RsIA_GT in *N. benthamiana*
15	RsIA_GT-SP-F	ATGTTATTGGCCCTTATTCTTACCA	Forward primer for signal peptide validation of RsIA_GT in yeast
16	RsIA_GT-SP-R	GGAGGGTGAAAGTACCACGAGC	Reverse primer for signal peptide validation of RsIA_GT in yeast
17	RsIA_GT-Mu-F	GGCGAAGCCATGCTTATGGTCGACGCCGACG GTGCTTCGAAATTCTCCGCCCTTGCCAAGCTC	Forward primer for analysis the cell-death suppressing ability of RsIA_GT independent of its enzyme activity in *N. benthamiana*
18	RsIA_GT-Mu-R	GAGCTTGGCAAGGGCGGAGAATTTCGAAGCA	Reverse primer for analysis the

	CCGTCGGCGTCGACCATAAGCATGGCTTCGCC	cell-death suppressing ability of RsIA_GT independent of its enzyme activity in *N. benthamiana*
